# Supplementary material for: DMRforPairs: identifying Differentially Methylated Regions between unique samples using array based methylation profiles
Source: BMC Bioinformatics. 2014 May 15;15:141. doi: 10.1186/1471-2105-15-141 (PMC4046028; doi:10.1186/1471-2105-15-141)
Supplement: Additional file 2 — DMRforPairs output for the comparison of A431-MCF7 and NA17018-NA17105. Please start from the HTML files in each folder. Available via the BMC Bioinformatics website. [file 1471-2105-15-141-S2.zip › 1394847754114233_MOESM2_ESM/A431_MCF7/figures/12460.pdf]

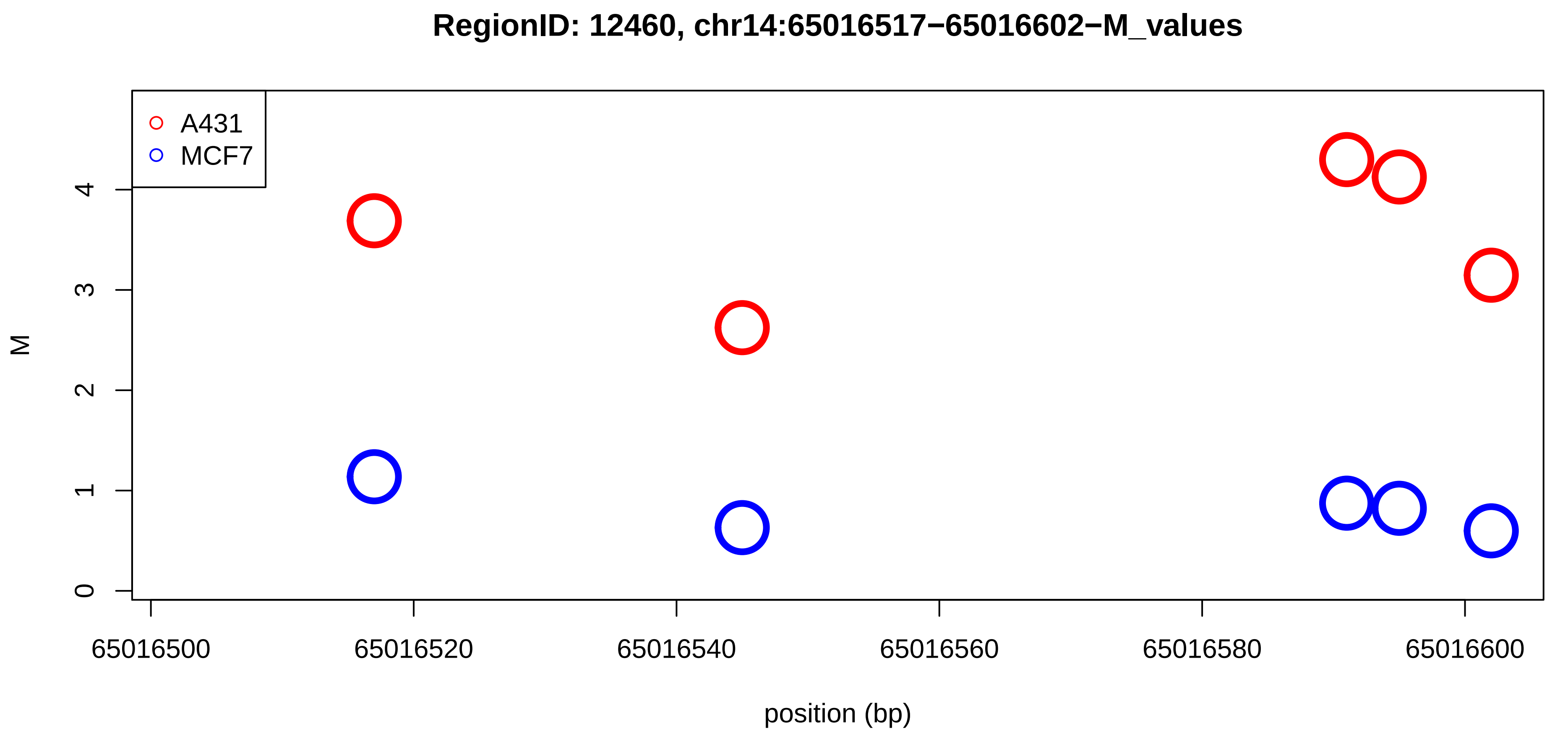
RegionID: 12460, chr14:65016517−65016602−M_values
A431
MCF7
M
0
1
2
3
4
65016500
65016520
65016540
65016560
65016580
65016600
position (bp)

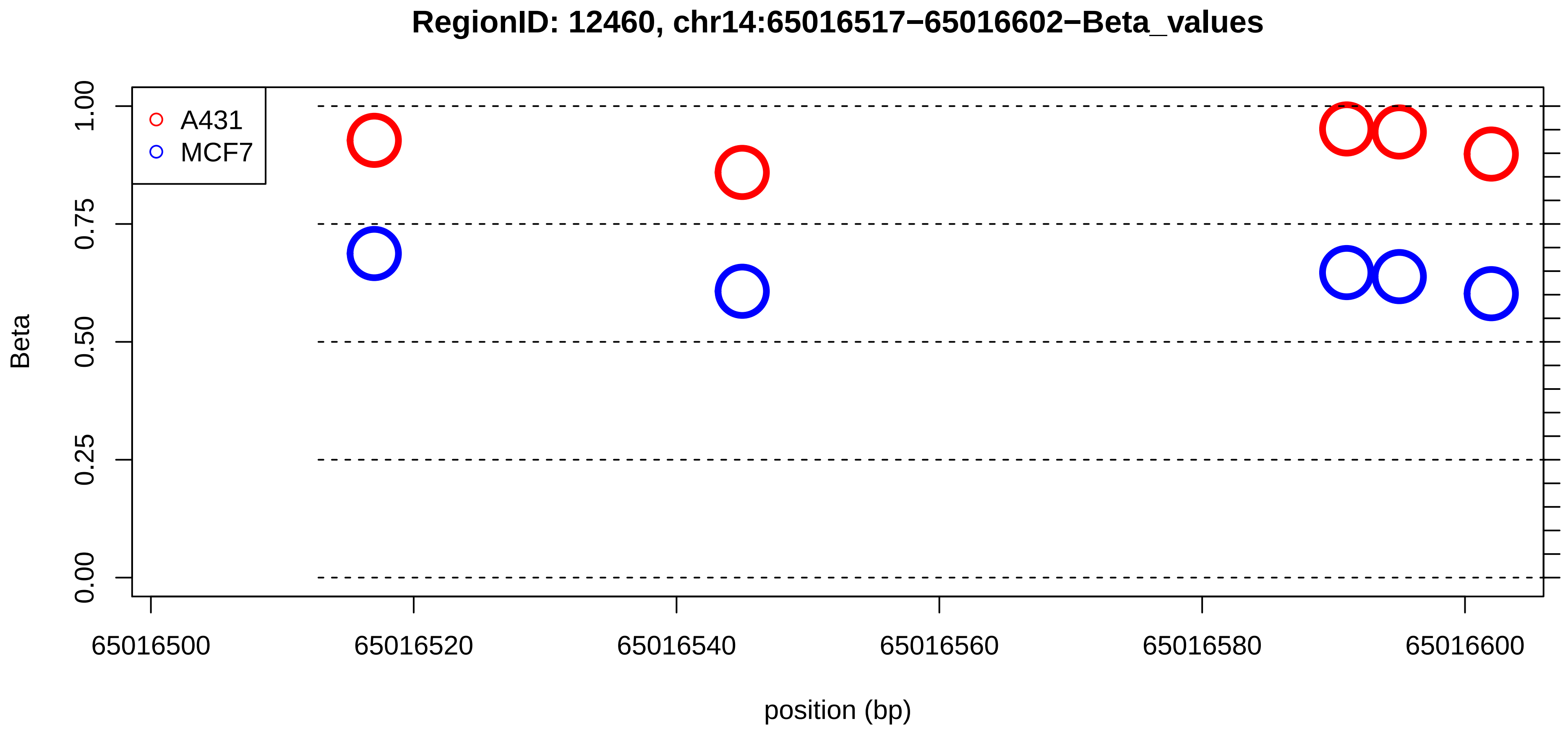
RegionID: 12460, chr14:65016517−65016602−Beta_values
A431
MCF7
Beta
0.00
0.25
0.50
0.75
1.00
65016500
65016520
65016540
65016560
65016580
65016600
position (bp)

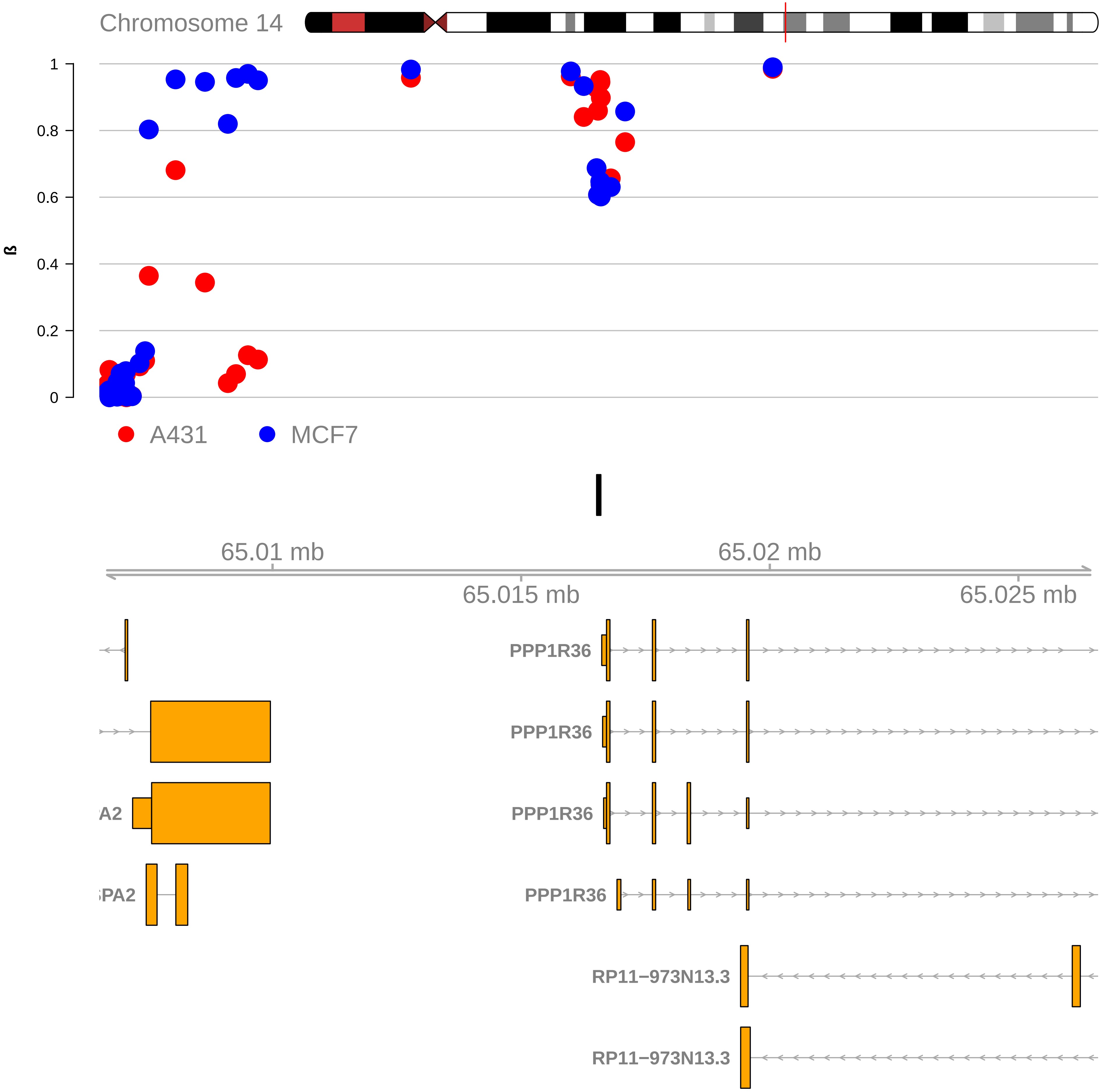

Chromosome 14
1
0.8
0.6
0.4
0.2
0
ß
A431
MCF7
65.01 mb
65.02 mb
65.015 mb
65.025 mb
PPP1R36
PPP1R36
A2
PPP1R36
PA2
PPP1R36
RP11−973N13.3
RP11−973N13.3
